# QUALITY OF LIFE ASSOCIATED WITH HOSPITAL DEATH AMONG PERSONS LIVING WITH DEMENTIA

**DOI:** 10.1093/geroni/igad104.2758

**Published:** 2023-12-21

**Authors:** Suzanne Sullivan, Chin-Shang Li, Yu-Ping Chang

**Affiliations:** SUNY Upstate Medical University, Syracuse, New York, United States; The State University of New York, University at Buffalo, Buffalo, New York, United States; University at Buffalo, The State University of New York, Buffalo, New York, United States

## Abstract

Dementia is a progressive, terminal health condition impacting quality-of-life. Hospice programs supporting family caregivers in managing end-of-life care for persons living with dementia (PLWD) and preventing non-beneficial hospitalizations are underutilized. This univariable logistic regression of the National Health and Aging Trends Study rounds 1-9 (n=719) aimed to identify associations between quality-of-life of PLWD and hospital death (yes/no). Results indicate that PLWD going outside most days (OR=3.34, p=0.0004) were more likely to die in a hospital compared to those never going outside. Those whose health did not prevent them from enjoying life (OR=2.5, p< 0.0001) were more likely to die in a hospital. PLWD needing >30 minutes to fall asleep (OR=2.43, p=0.002) compared to those never needing >30 minutes to fall asleep, PLWD without problems speaking (OR=1.69, p=0.007), not needing help to eat (OR=1.82, p=0.001), without problems chewing or swallowing (OR=1.54, p=0.039), and having possible dementia (OR=2.35, p< 0.0001) were more likely to die in the hospital. African Americans (OR=1.80, p=0.003) were more likely to die in the hospital compared to Whites, PLWD who received gas energy assistance (OR=1.95, p=0.048), and those living in the Pacific West compared to the Midwest or Northeast (OR=2.57, p=0.004) were more likely to die in the hospital. These findings suggest that quality-of-life, availability of support, financial and psychosocial well-being, and social disparities were associated with higher risk of hospital death among PLWD. Quality-of-life and psycho-social demographic features may serve as mechanistic targets for interventions supporting hospice transitions and prevention of non-beneficial end-of-life hospitalizations.

